# Fluoride ion adsorption isotherms, kinetics, and thermodynamics on iron(III) oxyhydroxide powders containing cellulose nanofibrils

**DOI:** 10.1007/s11356-023-25679-1

**Published:** 2023-02-08

**Authors:** Masahiro Umehara, Yoshiaki Kumamoto, Kenta Mukai, Akira Isogai

**Affiliations:** 1 Department of Biomaterials Science, Graduate School of Agricultural and Life Sciences, The University of Tokyo, Tokyo, 113-8657 Japan; 2 Research Center, Kao Corporation, 2-1-3 Bunka, Sumida-ku, 131-8501 Tokyo, Japan

**Keywords:** Adsorption kinetics, Adsorption thermodynamics, Diffusion model, Fluoride adsorption, Cellulose nanofibril, Iron oxyhydroxide, Langmuir isotherm

## Abstract

The adsorption isotherms, kinetics, and thermodynamics of fluoride ions (F^−^) on FeOOH powders in water were investigated to obtain fundamental information on FeOOH powders, which are used as F^−^ adsorbents in drinking and industrial water, and industrial wastewater. FeOOH powders were prepared as precipitates by mixing aqueous FeCl_3_ and NaOH solutions (1:3 mol/mol) in the presence of 2,2,6,6,-tetramethylpiperidine-1-oxyl radical (TEMPO)-oxidized cellulose nanofibrils (TOCNs), carboxymethylcellulose (CMC), or TEMPO-oxidized cellulose (TOC) fibers (without nanofibrillation), and subsequent drying and pulverizing. The FeOOH:TOCN, FeOOH:CMC, and FeOOH:TOC dry mass ratios were controlled at 87:13. The amount of F^−^ adsorbed by the FeOOH/TOCN powder per FeOOH mass was higher than those adsorbed by FeOOH, FeOOH/CMC, or FeOOH/TOC. The F^−^ adsorption isotherms on the FeOOH-containing powders showed higher correlation coefficients with the Langmuir model than with the Freundlich model. This indicates that F^−^ adsorbed on FeOOH initially formed a monolayer, predominantly via physical adsorption. Pseudo-second-order kinetics fitted well to the time-dependent F^−^ adsorption behaviors on the FeOOH-containing powders. Thermodynamic analysis of F^−^ adsorption on the FeOOH-containing powders showed that the Δ*G* values were negative, which indicates that F^−^ adsorption on the FeOOH-containing powders proceeded spontaneously in water. The negative Δ*G* value for FeOOH/TOCN was higher than those for FeOOH, FeOOH/CMC, and FeOOH/TOC at the same temperature. This shows that the FeOOH/TOCN powder can be used as an excellent and efficient F^−^ adsorbent in water.

**Graphical abstract:**

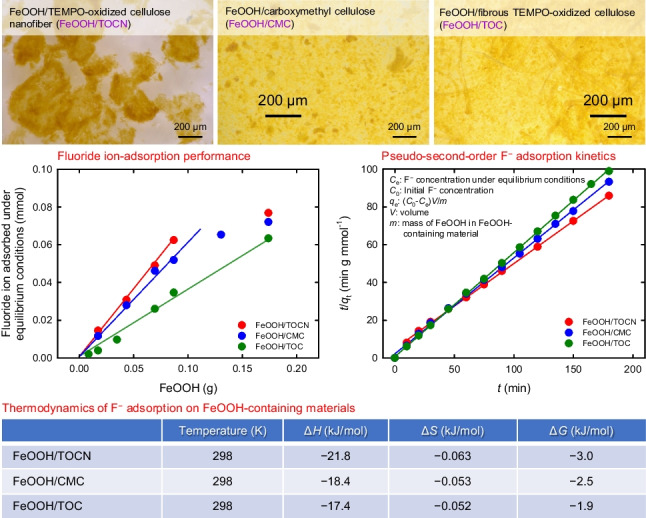

**Supplementary Information:**

The online version contains supplementary material available at 10.1007/s11356-023-25679-1.

## Introduction

The fluoride ion (F^−^) concentration in groundwater reaches the upper safety limit in some areas. The F^−^ concentrations in water must therefore be decreased to less than the concentration stipulated in regulations. Calcium hydroxide, calcium chloride, aluminum sulfate, polyaluminum chloride, and other aluminum compounds have been investigated as F^−^ adsorbents in water. However, the adsorption efficiencies of these compounds are insufficient (Asada and Eto 2000; Fukuta et al. 2004). Various metal oxides, metal ions, metal compounds, and inorganic clays, with or without biomass materials, have been reported as F^−^ adsorbents for decreasing the toxicity of drinking water. Metal–organic frameworks (MOFs) have also been proposed as highly selective F^−^ adsorbents (He et al. 2020; Jeyaseelan and Viswanathan 2021, 2022; Tang et al. 2022; Prabhu et al. 2021), although the use of MOFs for treating drinking water is expensive.

We reported in a previous paper (Umehara et al. 2022) that dried iron(III) oxyhydroxide (FeOOH) powders, which were obtained as water-insoluble precipitates by mixing aqueous FeCl_3_ and NaOH solutions, efficiently removed F^−^ from water. Use of a cellulose nanofibril/water dispersion in the preparation of the FeOOH precipitate improved the F^−^ adsorption efficiency compared with those of a commercially available FeOOH powder or an FeOOH powder synthesized without cellulose nanofibrils (Umehara et al. 2022). Here, cellulose nanofibrils were prepared from wood cellulose fibers by 2,2,6,6-tetramethylpiperidine-1-oxyl radical (TEMPO)-mediated oxidation and subsequent mechanical disintegration in water (Isogai 2018, 2021; Isogai et al. 2018).

The highest F^−^ adsorption efficiency was achieved by using an FeOOH:TEMPO-oxidized cellulose nanofibril (TOCN) mass ratio of 87:13. The F^−^ adsorption capability was stable over the pH range 4‒10. The F^−^ in the water partly replaced the Cl^−^ slightly present in the original the powder, which occurred by ion-exchange. However, the reason for the improvement in the F^−^ adsorption efficiency caused by the cellulose nanofibrils in the FeOOH powders was unclear. The abundant carboxylate groups present on the nanofibril surfaces and/or crystalline nanofibrils of width ~ 3 nm may have caused the formation of characteristic surface structures and/or morphologies of FeOOH/TOCN particles suitable for the efficient F^−^ adsorption.

In this study, FeOOH powders were synthesized with or without TOCNs, a water-soluble carboxymethylcellulose sodium salt (CMC), or fibrous TEMPO-oxidized cellulose (TOC). The TOCN and TOC samples had the same sodium carboxylate contents. However, the TOCNs had homogeneous fibril widths of ~ 3 nm, whereas the TOC had fiber widths of ~ 30 µm; the TOCN width was approximately 1/10 000 that of the TOC (Isogai and Zhou 2019).

The FeOOH-containing precipitates were dried and pulverized to fine powders. The F^−^ adsorption behaviors on the FeOOH, FeOOH/TOCN, FeOOH/CMC, and FeOOH/TOC powders were investigated under various conditions. The obtained experimental data were used to investigate the F^−^ adsorption isotherms, kinetics, and thermodynamics of the FeOOH-containing powders, on the basis of the data reported for other adsorbents (Hall et al. 1966; Annadurai et al. 2002; Srivastava et al. 2006; Hameed et al. 2007; Alkan et al. 2007; Tan et al. 2009; Foo and Hameed 2010; Liu et al. 2010; Dowood and Sen 2012; Tran et al. 2017). The obtained fundamental and scientific information will be useful in the development of FeOOH-containing powders for use as efficient adsorbents for F^−^ removal from drinking water.

## Materials and methods

### Materials

A commercial water-soluble CMC with a degree of substitution of 0.5–0.8, which corresponds to sodium carboxylate contents of 2.5–3.5 mmol/g, was used (Tokyo Chemical Ind. Co., Ltd., Tokyo, Japan). The CMC was completely dissolved in water by stirring a CMC/water mixture with a magnetic stirring bar at room temperature for more than 1 d. TEMPO-mediated oxidation was performed on a commercial softwood bleached kraft pulp with catalytic amounts of TEMPO and NaBr, and NaOCl as the primary oxidant, in water at pH 10 (Shinoda et al. 2012; Umehara et al. 2022). The TOC fibers were washed thoroughly with water by filtration. The obtained fibrous TOC had a sodium carboxylate content of 1.3 mmol/g and was stored at 4 °C in the wet state. The wet fibrous TOC was mechanically disintegrated in water with a blender to prepare a 1% (w/w) TOCN dispersion (Umehara et al. 2022). The obtained TOCN/water dispersion was highly viscous and transparent. FeCl_3_·6H_2_O, TEMPO, NaBr, NaOCl solution, and standard NaOH and HCl solutions were commercial products (Fujifilm Wako Chemical Ind. Ltd., Tokyo, Japan) and used as received. A commercial 2.4 mM NaF standard solution (TOA DKK Co., Ltd., Tokyo, Japan) was used in the F^−^ adsorption experiments.

### Preparation of FeOOH, FeOOH/TOCN, FeOOH/CMC, and FeOOH/TOC powders

Detailed procedures for the preparation of 100% FeOOH and FeOOH/TOCN composites with a FeOOH:TOCN dry mass ratio of 87:13 are available in a previous paper (Umehara et al. 2022). An FeCl_3_-containing solution (150 g, 0.05 mol) was slowly added under stirring to a 0.8% (w/w) CMC/water solution (83 g). Aqueous NaOH (150 g) containing 0.15 mol NaOH was then added to the FeCl_3_/CMC solution under stirring; the FeCl_3_:NaOH molar ratio was adjusted to 1:3. The mixture was stirred at room temperature for 12 h. The reddish FeOOH/CMC precipitate with a FeOOH:CMC dry mass ratio of 87:13 present in the mixture was separated and washed thoroughly with water by filtration. Similarly, an FeOOH/TOC precipitate with a FeOOH:TOC dry mass ratio of 87:13 was prepared from a fibrous TOC/water slurry by addition of aqueous FeCl_3_ and NaOH solutions. The precipitate was separated and washed with water by filtration. The mass recovery ratios for the FeOOH/TOCN, FeOOH/CMC, and FeOOH/TOC precipitates were ~ 100% based on the theoretical values. The precipitates were oven-dried at 65 °C for 12 h and then pulverized into fine powders as described in a previous paper (Umehara et al. 2022) for preparation of FeOOH, FeOOH/TOCN, FeOOH/CMC, and FeOOH/TOC powder samples.

### Analyses

The particle size distributions, Brunauer–Emmett–Teller (BET) surface areas, and Barrett–Joyner–Halenda (BJH) pore volumes of the FeOOH-containing powders were determined by using an electromagnetic vibrator sieve with various mesh sizes (AS200, Retsch, Germany) and a BELSorp mini II-type instrument (MicrotracBEL, Japan), respectively (Umehara et al. 2022). The FeOOH-containing powder (0–1 g) was added to a 2.4 mM NaF standard solution (40 mL) at 5, 25, or 40 °C under stirring at 400 rpm. The F^−^ concentration in the solution was measured with a fluoride electrode (F-2021 type, TOA DKK Co., Tokyo, Japan). The average quantity of F^−^ (mmol) adsorbed on the FeOOH-containing powder was obtained by performing three measurements in each experiment, and normalizing the values with respect to an FeOOH-containing powder with the same Fe content.

### Adsorption isotherms

The F^−^ concentration in the initial stage, *C*_0_ (2.4 mmol/L), and that under equilibrium conditions, *C*_*e*_ (mmol/L), after addition of the FeOOH-containing powder were determined in each experiment. The amount of F^−^ adsorbed on the FeOOH-containing powder per mass of FeOOH under equilibrium conditions, *q*_e_ (mmol/g), is expressed by Eq. (1):

1 $${q}_{e}=\frac{\left({C}_{0}-{C}_{e}\right)V}{m}$$

where *m* is the mass (g) of FeOOH in the FeOOH-containing powder and *V* (L) is the volume of the solution (*V* = 0.04 L in this study).

Four equations have been proposed to represent Langmuir isotherms under equilibrium adsorption conditions (see the electronic supplementary material). In this study, Eq. (2) (the Hanes–Woolf equation) was selected because straight lines with high correlation coefficients (*r*^2^) were obtained by using this equation (Mohammadi et al. 2012; Ahmadi et al. 2019). The data were somewhat scattered with low *r*^2^ values when the other three equations (the Lineweaver–Burk, Eadie–Hofstee, and Scatchard equations) were used to represent the Langmuir equilibrium isotherms (see the electronic supplementary material).

2 $$\frac{{C}_{e}}{{q}_{e}}=\left(\frac{1}{{Q}_{max}^{0}}\right){C}_{e}+\frac{1}{{Q}_{max}^{0}b}$$

where *Q*^0^_max_ (mmol/g) is the maximum amount of F^−^ adsorbed on the FeOOH-containing powder, forming *a* monolayer on the powder surface, and *b* is the Langmuir constant corresponding to the adsorption energy (Bothwall and Walker 1995; Ho and McKay 1998a, b, c; Annadurai et al. 2002; Vadivelan and Kumar 2005; Febrianto et al. 2009). The values of *Q*^0^_max_ and *b* were obtained from the slope and intercept, respectively, of the plot of *C*_*e*_/*q*_*e*_ against *C*_*e*_.

Another adsorption isotherm equation, namely, the Freundlich adsorption isotherm equation, is shown in Eq. (3):

3 $$\mathrm{ln}({q}_{e})=\mathrm{ln}{(K}_{f})+\frac{1}{n}\mathrm{ln}({C}_{e})$$

where *K*_*f*_ (mmol/g) is related to the adsorption ability, and 1/*n* shows the adsorption strength (Kumar et al. 2003; Wu et al. 2005; Liang et al. 2010; Xu and Li 2010; Zhao et al. 2013).

### Adsorption kinetics

The fist-order adsorption equation can be derived from the amount of F^−^ adsorbed on the FeOOH-containing powder per mass of FeOOH, *q*_*t*_ (mmol/g), at a certain time *t* (min) after addition of the powder to the NaF solution, and *q*_*e*_ (mmol/g) by using Eq. (4) (Ho and McKay 1998a, b, c; Azizian 2004; Vadivelan and Kumar 2005; Simonin 2016; Gupta and Bhattacharyya 2011):

4 $$\mathrm{ln}({q}_{e}-{q}_{t})=\mathrm{ln}({q}_{t})-(\frac{{k}_{1}}{2.303})t$$

where *k*_1_ is the adsorption rate constant.

The adsorption equation according to the pseudo-second-order model is shown in Eq. (5) (Ho and McKay 1998a, b, c; Azizian 2004; Ho 2006a, b),

5 $$\frac{t}{{q}_{t}}=\frac{1}{{k}_{2}{q}_{e}^{2}}+\frac{t}{{q}_{t}}$$

where 1/*k*_2_ is the adsorption rate constant.

When a chemosorption effect contributes dynamically to F^−^ adsorption on the heterogeneous solid surface of an adsorbent as a secondary factor, the Elovich equation (Eq. (6)) is applicable (Ho and McKay 1998a, b, c; Ho 2006a, b; Wu et al. 2009):

6 $${q}_{t}=\frac{1}{\beta }\mathrm{ln}\left(t\right)+\frac{1}{\beta }\mathrm{ln}(\alpha \beta )$$

where *α* is the initial adsorption rate, and *β* is the detachment rate constant or the constant related to an activation energy arising from chemisorption.

### Diffusion models

The process of F^−^ diffusion into pores in the FeOOH-containing powders can be investigated by using an intraparticle diffusion model (Weber and Morris 1963; McKay and Poots 1980) and the Bangham diffusion model (Bhatnagar and Jain 2004; Kumar et al 2009; Kannan and Sundaram 2001; Kavitha and Namasivayam 2007), i.e., Eqs. (7) and (8), respectively:

7 $${q}_{t}={K}_{p}{t}^\frac{1}{2}+C$$

8 $$\ln(\ln\left(\frac{C_0}{C_0-q_tm}\right))=\ln\left(\frac{k_0m}{2.303V}\right)+\alpha\;\ln\;(t)$$

where *K*_*p*_ (mmol/L/min^1/2^) is the intraparticle rate constant, and *K*_0_ is the Bangham rate constant for diffusion into fine pores.

### Adsorption thermodynamics

The values of *q*_*e*_ and *C*_*e*_ were determined in NaF solutions at 5, 25, and 40 °C in the presence of the FeOOH-containing powder. The thermodynamic parameters Δ*G*, Δ*H*, and Δ*S* were obtained from *q*_*e*_ and *C*_*e*_ at the three temperatures *T* (K) according to Eq. (9),

9 $$\mathrm{ln}\frac{{q}_{e}}{{C}_{e}}=-\frac{\Delta G}{RT}=-\frac{\Delta H}{RT}+\frac{\Delta S}{R}$$

## Results and discussion

### Preparation and characterization of FeOOH-containing powders

**Fig. 1:**
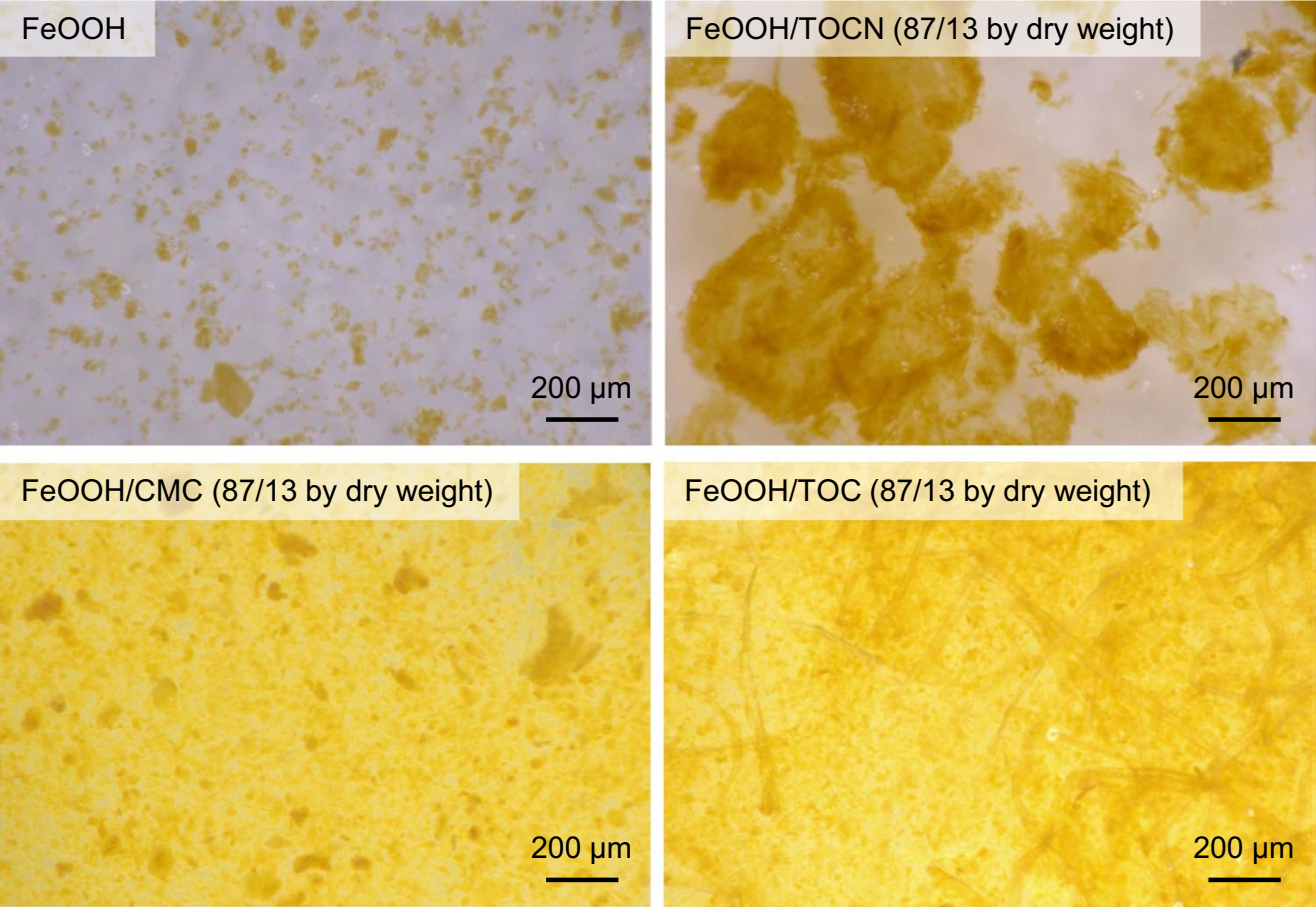
Optical microscopy images of FeOOH-containing powders

In our previous study, the amount of F^−^ adsorbed by the FeOOH/TOCN powder with a FeOOH:TOCN dry mass ratio of 87:13 was higher than the amounts adsorbed by other FeOOH/TOCN powders with different FeOOH:TOCN dry mass ratios. In this study, FeOOH/CMC and FeOOH/TOC powders with 87:13 dry mass ratios were prepared. Their optical microscopy images, and BET/BJH parameters and average sizes, along with those of 100% FeOOH, are shown in Fig. 1 and Table 1, respectively.

**Table 1:** Surface and pore parameters and average particle sizes of FeOOH-containing powders

Sample	BET specific surface area (m^2^/g)^a^	BJH total pore volume (mL/g)^a^	BJH average pore diameter (nm)^a^	Size-average particle size (µm)^b^
FeOOH^c^	79	0.09	1.9	93
FeOOH/TOCN^c^ (87:13 by dry mass)	106	0.06	2.2	188
FeOOH/CMC (87:13 by dry mass)	92	0.05	2.1	107
FeOOH/TOC (87:13 by dry mass)	92	0.05	2.1	145

^a^Determined by N_2_-adsorption isotherm method

^b^Measured by vibrator sieve method

^c^Umehara et al. (2022)

The order of the average particle sizes was FeOOH/TOCN > FeOOH/TOC > FeOOH/CMC > FeOOH (Table 1); this is in good agreement with the microscopy images (Fig. 1). Figure 1 shows that the FeOOH/TOC powder contained TOC fibers; therefore, the average particle size of this sample was larger than those of FeOOH and FeOOH/CMC. The BET specific surface areas and BJH total pore volumes can affect F^−^ adsorption on the FeOOH-containing powders when physical adsorption of F^−^ is predominant rather than chemosorption, e.g., via ionic-bond formation between F^−^ and the FeOOH surfaces.

### Adsorption isotherms of F^−^ on FeOOH-containing powders

**Fig. 2:**
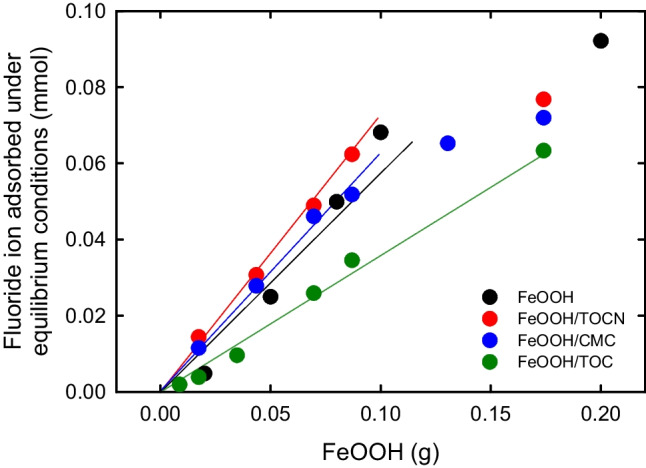
Relationship between FeOOH mass in FeOOH-containing powders and amount of F^−^ adsorbed on each FeOOH-containing powder under equilibrium conditions

The relationship between the amount of F^−^ adsorbed on each FeOOH-containing powder under equilibrium conditions and the FeOOH mass in the powder was determined by using the procedure described in the Materials and Methods section; the results are shown in Fig. 2. When the FeOOH mass was less than 0.1 g, the amount of F^−^ adsorbed per FeOOH mass was highest for FeOOH/TOCN and then FeOOH/CMC and FeOOH. The FeOOH/TOC sample gave the lowest amount of F^−^ adsorbed per FeOOH mass. These results can be explained by FeOOH/TOCN having the largest BET specific surface area and average particle size (Table 1).

**Fig. 3:**
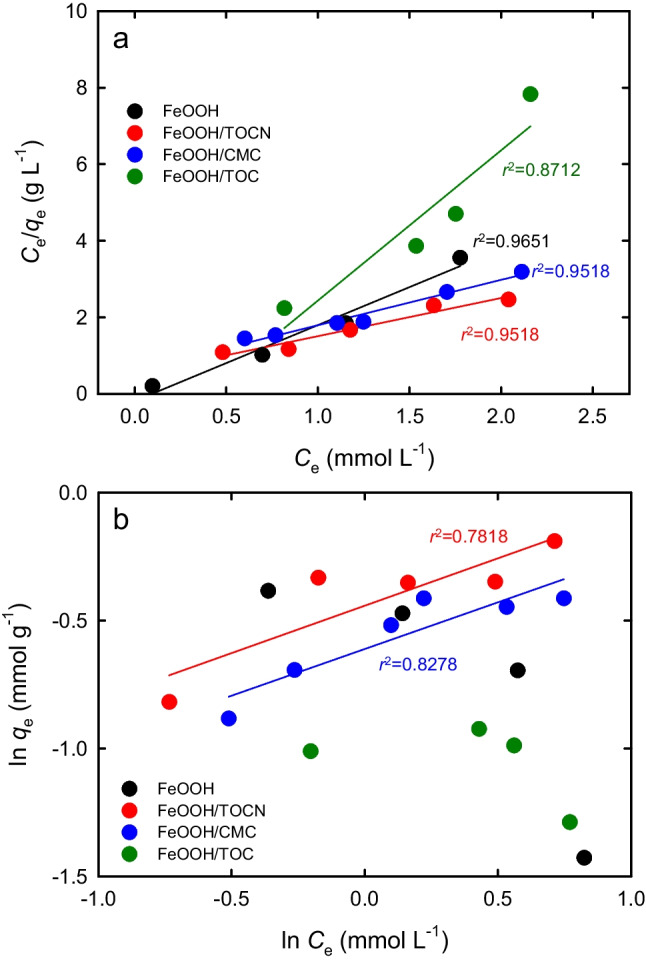
F^−^ adsorption isotherms on FeOOH-containing powders based on (**a**) Langmuir and (**b**) Freundlich models

Equations (1)–(3) were used to investigate the adsorption behaviors of F^−^ on the FeOOH-containing powders under equilibrium conditions in terms of the Langmuir and Freundlich isotherm models; the results are shown in Fig. 3. In the case of the Langmuir adsorption isotherm, the other three equations that have been proposed are described in the electronic supplementary material. However, the data obtained with these three equations were somewhat scattered and showed lower *r*^2^ values (Figs. S1-S3 in the electronic supplementary material) than those in Fig. 3a. Equation (2) was therefore selected for this study.

Straight lines were obtained from the plots for each powder by using the least-squares method; high *r*^2^ values, i.e., 0.95–0.97, were obtained for FeOOH, FeOOH/TOCN, and FeOOH/CMC (Fig. 3a). In contrast, the plots based on the Freundlich isotherm model, which were obtained using Eq. (3), were rather scattered (Fig. 3b), and the *r*^2^ value for each powder was lower than that in Fig. 3a. These results show that F^−^ adsorption on the FeOOH-containing powders predominantly followed the Langmuir model, and the F^−^ adsorbed on the surfaces of the FeOOH-containing powders initially formed a monolayer. When multilayer adsorption of F^−^ occurs on the powder surface, the obtained data fit the Freundlich adsorption isotherm model. Table S1 in the electronic supplementary material lists some F^−^ adsorbents that have been reported in the literature. Most of the samples in Table S1 show Langmuir adsorption isotherms, although a few of them show Freundlich adsorption isotherms.

The values of the equilibrium parameter constant *R*_*L*_ were obtained from Eq. (10) by using the values *b* and *C*_0_ in Eq. (2). The *R*_*L*_ value indicates whether an adsorption is favorable or unfavorable (Annadurai et al. 2002; Liu 2009)

10 $${R}_{L}=\frac{1}{1+b{C}_{0}}$$

The *R*_*L*_ values were 0.07, 0.40, 0.04, and 0.40 for FeOOH, FeOOH/TOCN, FeOOH/CMC, and FeOOH/TOC, respectively. All these values were in the range 0–1, which shows that these FeOOH-containing powders are favorable F^−^ adsorbents.

### Adsorption kinetics of F^−^ on FeOOH-containing powders

**Fig. 4:**
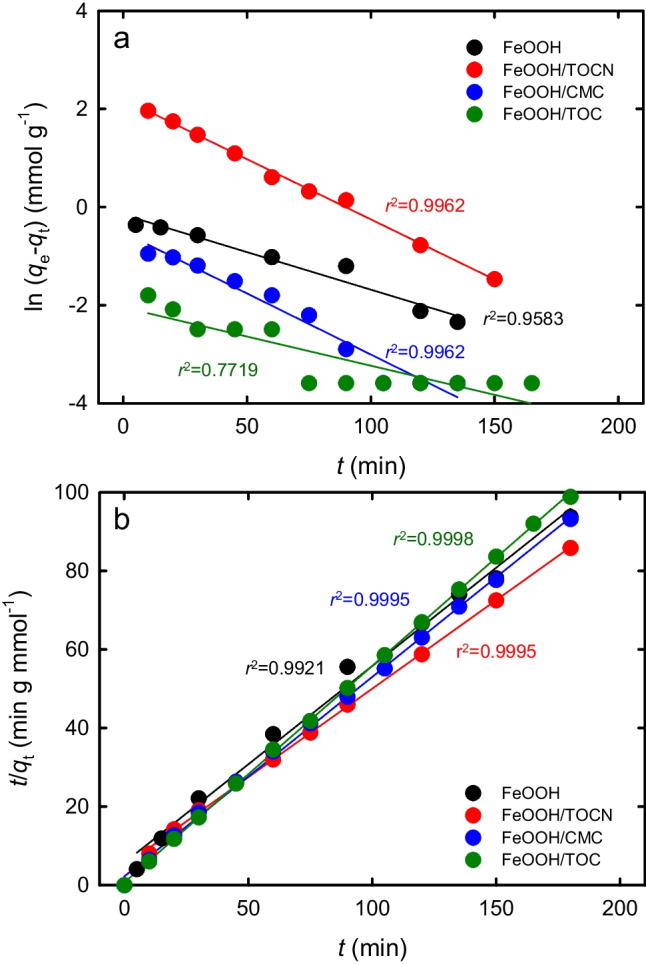
F^−^ adsorption kinetics on FeOOH-containing powders based on (**a**) pseudo-first-order and (**b**) pseudo-second-order models

The *q*_*t*_ values at a mixing time *t* (min) after addition of the FeOOH-containing powders to the NaF solution were determined. The results were plotted according to Eqs. (4) and (5), which are based on the pseudo-first-order and pseudo-second-order adsorption models, respectively (Fig. 4a and b, respectively). The *r*^2^ value for each FeOOH-containing powder was higher for the pseudo-second-order model (Fig. 4b) than that for the pseudo-first-order model (Fig. 4a). This shows that the F^−^ adsorption/desorption interactions occurred on the FeOOH surfaces (Kumar et al. 2009; Zhang et al. 2019; Mahmound et al.^ 2020^).

Using Eq. (5), the kinetic constants 1/*k*_2_ and the *q*_*e*_ values under equilibrium conditions were calculated from the plots in Fig. 4b; the results are listed in Table 2. The orders of the 1/*k*_2_ and *q*_*e*_ values were FeOOH/TOCN > FeOOH > FeOOH/CMC > FeOOH/TOC and FeOOH/TOCN > FeOOH = FeOOH/CMC > FeOOH/TOC, respectively. The FeOOH/TOCN powder therefore gave the highest adsorption rate and the highest adsorbed amount of F^−^ per mass of FeOOH in the powder. These results can be explained by FeOOH/TOCN having the largest BET specific surface area and average particle size (Table 1).

**Table 2:** Values of 1/*k*_2_ and *q*_*e*_ obtained from the results in Fig. 4b by using Eq. (5)

Sample	1/*k*_2_ (mmol/g/min)	*q*_e_ (mmol/g)	Sample	1/*k*_2_ (mmol/g/min)	*q*_e_ (mmol/g)
FeOOH	10.1	17	FeOOH/TOCN	10.7	19
FeOOH/CMC	5.3	17	FeOOH/TOC	1.0	15

The F^−^ adsorption kinetics reported in the literature for various adsorbents are listed in Table S2 in the electronic supplementary material. The data for most of the adsorbents show that the pseudo-second-order model fits the kinetics of F^−^ adsorption well.

**Fig. 5:**
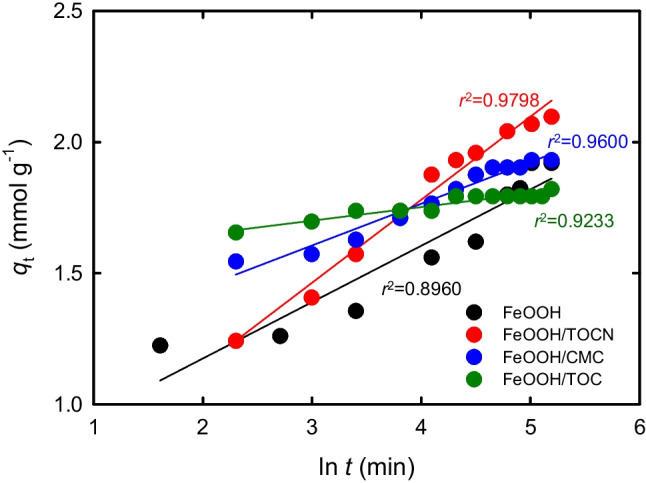
Elovich plots for amount of F^−^ adsorbed on FeOOH-containing powders

Elovich plots empirically indicate the contribution of chemisorption of F^−^ on the FeOOH-containing powders in addition to that of physical adsorption (Ho and McKay 1998a, b, c; Ho 2006a, b; Wu et al. 2009). Figure 5 shows Elovich plots obtained by using Eq. (6). However, the *r*^2^ value for each powder was lower than that for the same powder calculated by using the pseudo-second-order model (Fig. 4b). Chemisorption, e.g., via ionic-bond formation, therefore, did not significantly contribute to F^−^ adsorption on the FeOOH-containing powders. Physical adsorption predominantly affected F^−^ adsorption on the FeOOH-containing powders.

### Models of F^−^ diffusion into pores of FeOOH-containing powders

**Fig. 6:**
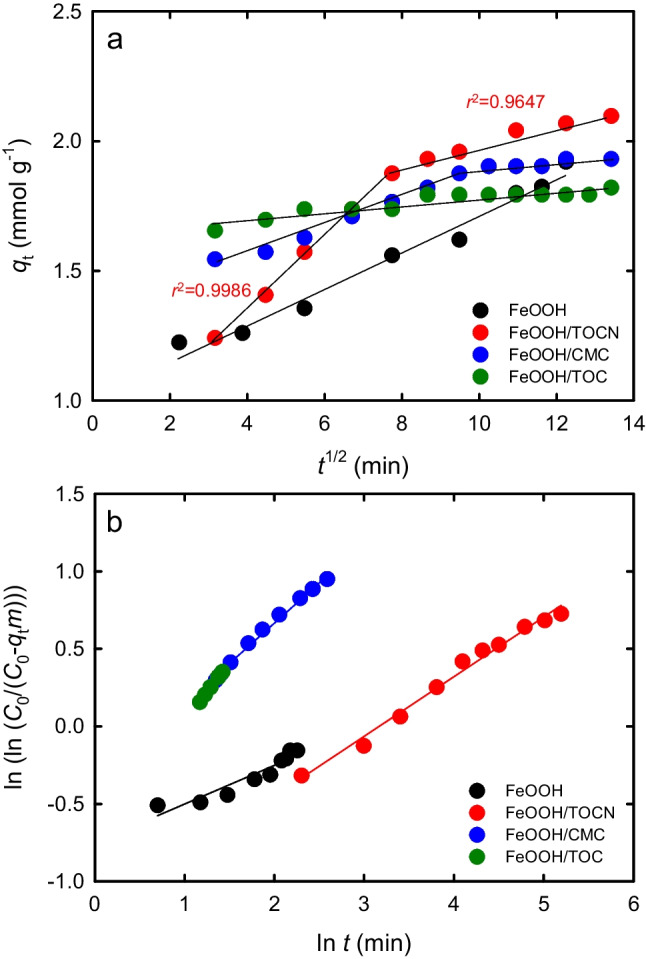
Time-dependent plots of amount of F^−^ adsorbed on FeOOH-containing powders, based on (**a**) intraparticle diffusion model and (**b**) Bangham diffusion model

The first-order or second-order adsorption models or the Elovich model cannot be used to clarify dynamic diffusion kinetics. However, the intraparticle diffusion model (Weber and Morris 1963; McKay and Poots 1980; Kannan and Sundaram 2001; Kavitha and Namasivayam 2007) or the Bangham model (Bhatnagar and Jain 2004; Kumar et al 2009) can provide some information on the kinetics of F^−^ diffusion into the pores of the FeOOH-containing powders. Plots for the intraparticle and Bangham diffusion models are shown in Fig. 6a and b, respectively.

Figure 6a shows that the plot against *t* for the FeOOH/TOCN powder indicated two slopes; the high adsorption rate constant decreased at *t* > 7.5 min. This indicates that F^−^ ions may have first diffused into macropores in FeOOH/TOCN at the high adsorption rate and then diffused into mesopores and micropores at the lower adsorption rate; two clear diffusion stages may have been involved in the F^−^ adsorption process. Comparable results were obtained for FeOOH/CMC, although the diffusion rate constants were lower than those for FeOOH/TOCN.

The constant *α* and the Bangham rate constant *k*_0_ for F^−^ diffusion into fine pores in the FeOOH-containing powders were obtained from the data in Fig. 6b by using Eq. (8). The *r*^2^ values were 0.90, 0.99, 0.95, and 0.92 for FeOOH, FeOOH/TOCN, FeOOH/CMC, and FeOOH/TOC, respectively, which shows high correlation. The values of *α* were less than 1 for all the powders. This suggests that after adsorption on the surface, the F^−^ ions may have diffused into the fine pores after a time lapse (Largitte and Pasquier 2016). The Bangham diffusion rate constants *k*_0_ were 3.3, 2.4, 5.2, and 8.2 for FeOOH, FeOOH/TOCN, FeOOH/CMC, and FeOOH/TOC, respectively. This shows that a higher diffusion rate constant resulted in a lower *q*_*e*_ value (Fig. 2). The reason for such observation needs further investigation in the future.

### Thermodynamics of F^−^ adsorption on FeOOH-containing powders

**Fig. 7:**
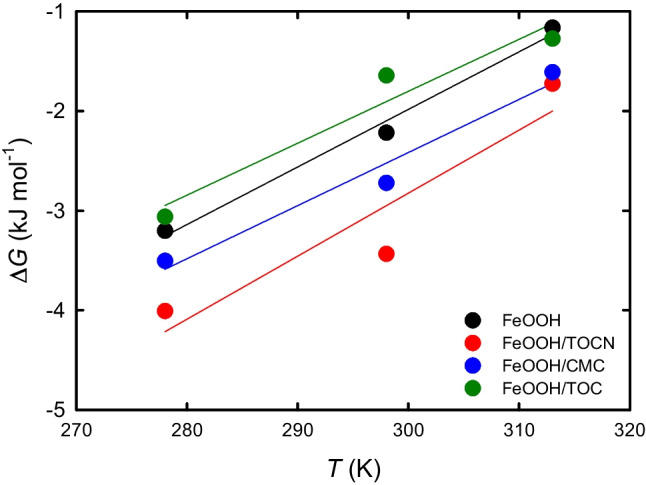
Relationships between temperature and Δ*G* for F^−^ adsorption on FeOOH-containing powders

Figure 7 shows the relationship between the temperature *T* (K) and Δ*G* [= ‒*RT*ln(*q*_e_/*C*_e_)] (Meenakshia et al. 2008; Zhao et al. 2010). The thermodynamic parameters of F^−^ adsorption on the FeOOH-containing powders were investigated by using Eq. (9) to calculate the values of Δ*H* and Δ*S* from the straight lines in Fig. 7. The obtained Δ*G*, Δ*H*, and Δ*S* values are listed in Table 3.

**Table 3:** Thermodynamic parameters for F^−^ adsorption on FeOOH-containing powders

Sample	*T* (K)	Δ*H* (KJ/mol)	Δ*S* (kJ/mol)	Δ*G* (kJ/mol)
FeOOH	278	‒19.3	‒0.058	‒3.2
	298			‒2.1
	313			‒1.2
FeOOH/TOCN (87/13 by dry mass)	278	‒21.8	‒0.063	‒4.2
	298			‒3.0
	313			‒2.0
FeOOH/CMC (87/13 by dry mass)	278	‒18.4	‒0.053	‒3.6
	298			‒2.5
	313			‒1.7
FeOOH/TOC (87/13 by dry mass)	278	–17.4	‒0.052	‒2.9
	298			‒1.9
	313			‒1.1

Negative Δ*G* values were obtained at three temperatures, which indicate that F^−^ adsorption on the FeOOH-containing powders proceeded spontaneously in solutions. All the Δ*H* values were negative; therefore, F^−^ adsorption on the FeOOH-containing powders was exothermic. The negative Δ*G* value decreased slightly with increasing temperature. This indicates that adsorption between F^−^ and the FeOOH-containing powders became weaker at high temperatures. This result supports the possibility that chemosorption, via covalent-like bonding or inner sphere complex formation, have made a minor contribution to F^−^ adsorption on the FeOOH-containing powders. F^−^ adsorption on the powders predominantly involved physical interactions.

## Conclusion

The amount of F^−^ adsorbed on an FeOOH/TOCN powder per FeOOH mass was higher than those adsorbed on FeOOH, FeOOH/CMC, or FeOOH/TOC. The FeOOH/TOCN powder can therefore be used as an efficient adsorbent for F^−^ removal form drinking and industrial water and industrial wastewater. The F^−^ adsorption isotherms on the FeOOH-containing powders showed higher correlation coefficients with the Langmuir model than those with the Freundlich model. This indicates that F^−^ adsorbed on FeOOH initially formed a monolayer, predominantly via physical adsorption. Pseudo-second-order kinetics well fitted the time-dependent F^−^ adsorption behaviors on the FeOOH-containing powders. The intraparticle and Banham diffusion models showed that the F^−^ ions first diffused into macropores in FeOOH/TOCN or FeOOH/CMC at a high adsorption rate and then diffused into mesopores and micropores at a lower adsorption rate. Thermodynamic analysis of F^−^ adsorption on the FeOOH-containing powders showed that the Δ*G* values were negative at 278, 298, and 313 K. This shows that F^−^ adsorption on the FeOOH-containing powders proceeded spontaneously in NaF solutions at these three temperatures. The negative Δ*G* value for FeOOH/TOCN was higher than those for FeOOH, FeOOH/CMC, and FeOOH/TOC at the same temperature. This shows that the FeOOH/TOCN powder is an excellent F^−^ adsorbent. Because of its nanoscale morphology, the FeOOH/TOCN powder had the largest specific surface area and average particle size. This is probably why it showed the most efficient F^−^ adsorption in water.

## Supplementary Information

Below is the link to the electronic supplementary material.

Supplementary file1 (DOCX 66 KB)

## Data Availability

The datasets used and/or analyzed during the current study are available from the corresponding author on reasonable request.
