# Supplementary material for: Fluoride ion adsorption isotherms, kinetics, and thermodynamics on iron(III) oxyhydroxide powders containing cellulose nanofibrils
Source: Environ Sci Pollut Res Int. 2023 Feb 8;30(16):48201–10. doi: 10.1007/s11356-023-25679-1 (PMC10097791; doi:10.1007/s11356-023-25679-1)
Supplement: Supplementary file 1 — Supplementary file1 (DOCX 66 KB) [file 11356_2023_25679_MOESM1_ESM.docx]

*Electronic Supplementary Material*

**Fluoride ion adsorption isotherms, kinetics, and thermodynamics on iron(III) oxyhydroxide powders containing cellulose nanofibrils**

Masahiro Umehara • Yoshiaki Kumamoto • Kenta Mukai • Akira Isogai

M. Umehara ⋅ Y. Kumamoto ⋅ K. Mukai

Research Center, Kao Corporation, 2-1-3 Bunka, Sumida-ku, Tokyo 131-8501, Japan

M. Umehara ⋅ A. Isogai (🖂)

Department of Biomaterials Science, Graduate School of Agricultural and Life Sciences, The University of Tokyo, Tokyo 113-8657, Japan

e-mail: [akira-isogai@g.ecc.u-tokyo.ac.jp](mailto:aisogai@mail.ecc.u-tokyo.ac.jp)

**Fig. S1** Langmuir plots of F^─^ adsorption on FeOOH-containing powders, according to equation reported by Kaufman and Jain (1992)

**Fig. S2** Langmuir plots of F^─^ adsorption on FeOOH-containing powders, according to equation reported by Mondal et al. (1993)

**Fig. S3** Langmuir plots of F^─^ adsorption on FeOOH-containing powders, according to equation reported by Crist et al. (1994)

**Table S1** F^─^ adsorption isotherms, Langmuir or Freundlich, on various adsorbents reported in the literature

| Absorbent | Isotherm | Reference |
| --- | --- | --- |
| FeOOH/TOCN | **Langmuir** | This study |
| Mg/Fe layered double hydroxides | **Langmuir** | Wu et al. (2015) |
| Amorphous aluminum hydroxide | **Langmuir** | Zhang et al. (2016) |
| Tea waste-loaded with Al/Fe oxides | **Langmuir** | Cai et al. (2015) |
| Iron oxide | **Langmuir** | Zhang et al. (2017) |
| Cerium dispersed in carbon | Freundlich | Sivasankara et al. (2013) |
| Zr(IV) impregnated dithiocarbamate-modified chitosan beads | Freundlich | Liu et al. (2013) |
| Al-doping chitosan/Fe(III) hydrogel | **Langmuir** | Ma et al. (2014) |
| Fe/Mg/La triple-metal composite | **Langmuir** | Yu et al. (2015) |
| Ag/Al/Zr triple-metal composite | Freundlich | Wang et al. (2017) |
| Indian sandalwood leaf powder | **Langmuir** | Khound and Bharali (2018) |
| GO/MgO/MgFe_2_O_4_ binary oxides | **Langmuir** | Sahoo and Hota (2018) |
| Zr(IV)-impregnated magnetic chitosan graphene oxide | **Langmuir** | Liu et al. (2021) |
| Fe_2_O_3_/areca nut-activated carbon composite | **Langmuir** | Joshi and Pradhananga (2016) |
| Nanomagnetite aggregated schwertmannite | **Langmuir** | Goswami and Purkait (2014) |
| FeOOH | **Langmuir** | Mohamed et al. (2017) |
| Fe-impregnated chitosan | **Langmuir** | Zhang et al. (2015) |
| Biochars | **Langmuir** | Kumar et al. (2019) |
| Ce@BTC MOFs | **Langmuir** | Jeyaseelan and Viswanathan (2021) |
| Hydroxyapatite-entrenched cerium MOFs | **Langmuir** | Jeyaseelan and Viswanathan (2022) |
| Carbon dots capped cerium oxide nanoparticles | Freundlich | Meng et al. (2022) |

**Table S2** F^─^ adsorption kinetics, first- or second-order, on various adsorbents reported in the literature

| Absorbent | Kinetic order | Reference |
| --- | --- | --- |
| FeOOH/TOCN | **Second** | This study |
| Hybrid lanthanum-carbon | **Second** | Vences-AlvarezaLitz et al. (2015) |
| CeCOOH | **Second** | Zhang et al. (2016) |
| Al(OH)_3_ | **Second** | Zhang and Jia (2016) |
| ZrO_2_ MOF | **Second** | He et al. (2016) |
| Al/hydroxyapatite | **Second** | He et al. (2017) |
| Hypae-supported alumina | **Second** | Yang et al. (20 (17) |
| MgO/AlO/ZrO_2_ | **Second** | Aihe et al. (2017) |
| Integrated Uio-66/CF hybrid | **Second** | Xie et al. (2019) |
| Cellulose/hydroxyapatite | **Second** | Yu et al. (2013) |
| Alginate/nano-hydroxyapatite | **Second** | Pandi and Viswanathan (2014) |
| La-impregnated cellulose | **Second** | Nagaraji et al. (2017) |
| Geomaterials | First | Sujana et al. (2019) |
| Manganese oxide/alumina | First | Teng et al. (2009) |
| Biocarbon/Mg/Al layered double hydroxides | **Second** | Ma et al. (2014) |
| Hydroxyapatite nanowires | **Second** | He et al. (2016) |
| Cucumis pubescens | **Second** | Kazi et al. (2018) |
| Rice spike-like akageneite anchored graphene oxide | **Second** | Iiu et al. (2016) |
| Calcite nanoparticle | **Second** | Budyanto et al. (2015) |
| Biochar | **Second** | Sadhu et al. (2022) |
| Layered double hydroxides | **Second** | Liu et al (2018) |

**References**

Ahamad KU, Singh R, Baruah I, Choudhury H, Sharm MR (2018) Equilibrium and kinetics modeling of fluoride adsorption onto activated alumina, alum and brick powder. Groundwater Sustain Develop 7:452‒458

https://doi.org/10.1016/j.gsd.2018.06.005

Aihe W, Kanggen Z, Xing L, Fang L, Chun Z, Quanzhou C (2017) Granular tri-metal oxide adsorbent for fluoride uptake: Adsorption kinetic and equilibrium studies. J Colloid Interf Sci 505:947‒955

https://doi.org/10.1016/j.jcis.2017.06.074

Budyanto SB, Kuo YL, Liu JC (2015) Adsorption and precipitation of fluoride on calcite nanoparticles: A spectroscopic study. Sep Purif Technol 150:325‒331

https://doi.org/10.1016/j.seppur.2015.07.016

Cai HM, Chen GJ, Peng CY, Zhang ZZ, Dong YY, Shang GZ, Zhu XH, Gao HJ, Wan XC (2015) Removal of fluoride from drinking water using tea waste loaded with Al/Fe oxides: A novel, safe and efficient biosorbent. Appl Surf Sci 328:34‒44

https://doi.org/10.1016/j.apsusc.2014.11.164

Crist RH, Martin JR, Carr D, Watson JR, Clarke HJ, Crist DLR (1994) Interaction of metals and protons with algae. 4. Ion-exchange vs adsorption models and a reassessment of Scatchard plots: Ion-exchange rates and equilibria compared with calcium alginate. Environ Sci Technol 28:1859‒1866

Ekuma FK, Chukwuemeka-Okorie HO, Okoyeagu A, Chimeziri CC (2019) Studies on the adsorption of tartrazie and sunset yellow dyes from aqueous solution using activated gbafilo (Chrysobalanus icaco) shell. J Chem Soc Nigeria 44:937‒947

https://journals.chemsociety.org.ng/index.php/jcsn/article/view/353/412

Goswami A, M. K. Purkait MK (2014) Removal of fluoride from drinking water using nanomagnetite aggregated schwertmannite. J Water Process Eng 1:91–100

https://doi.org/10.1016/j.jwpe.2014.03.009

He J, Cai X, Chen K, Li Y, Zhang K, Jin Z, Meng F, Liu N, Wang X, Kong L, Huang X, Liu J (2016) Performance of a novelly-defined zirconium metal-organic frameworks adsorption membrane in fluoride removal. J Colloid Interf Sci 484:162‒172

https://doi.org/10.1016/j.jcis.2016.08.074

He J, Zhang K, Wu S, Cai X, Chen K, Li Y, Sun B, Jia Y, Meng F, Jin Z, Kong L, Liu J (2016) Performance of novel hydroxyapatite nanowires in treatment of fluoride contaminated water. J Hazard Mater 303:119‒130

https://doi.org/10.1016/j.jhazmat.2015.10.028

He J, Chen K, Cai X, Li Y, Wang C, Zhang K, Jin Z, Meng F, Wang X, Kong L, Liu J (2017) A biocompatible and novelly-defined Al-HAP adsorption membrane for highly effective removal of fluoride from drinking water. J Colloid Interf Sci 490:97‒107

https://doi.org/10.1016/j.jcis.2016.11.009

Iiu Y, Lv J, Jin W, Zhao Y (2016) Defluoridation by rice spike-like akaganeite anchored graphene oxide. RSC Adv 6:11240‒11249

https://doi.org/10.1039/C5RA24565F

Jeyaseelan A, Viswanathan N (2021) Facile synthesis of tunable rare earth based metal organic frameworks for enhanced fluoride retention. J Mol Liq 326:115163

https://doi.org/10.1016/j.molliq.2020.115163

Jeyaseelan A, Viswanathan N (2022) Investigation of hydroxyapatite-entrenched cerium organic frameworks incorporating biopolymeric beads for efficient fluoride removal. Ind Eng Chem Res 61:7911‒7925

https://pubs.acs.org/doi/10.1021/acs.iecr.2c00487

Joshi S, Pradhananga MA(2016) Removal of fluoride ions by adsorption onto Fe_2_O_3_ /areca nut activated carbon composite. J I Eng 12:175‒183

https://doi.org/10.3126/jie.v12i1.16901

Kaufman EN, Jain RK (1992) Effect of bivalent interaction upon apparent antibody-affinity: Experimental confirmation of theory using fluorescence photobleaching and implications for antibody-binding assays. Cancer Res 52:4157‒4167

Kazi TG, Brahman KD, Baig JA, Afridi HI (2018) A new efficient indigenous material for simultaneous removal of fluoride and inorganic arsenic species from groundwater. J Hazard Mate 357:159‒167

https://doi.org/10.1016/j.jhazmat.2018.05.069

Khound NJ, Bharali RK (2018) Biosorption of fluoride from aqueous medium by Indian sandalwood (Santalum Album) leaf powder. J Environ Chem Eng 6:1726–1735

https://doi.org/10.1016/j.jece.2018.02.010

Kumar H, Patel M, Mohan D (2019) Simplified batch and fixed-bed design system for efficient and sustainable fluoride removal from water using slow pyrolyzed okra stem and black gram straw biochars. ACS Omega 4:19513‒19525

https://doi.org/10.1021/acsomega.9b00877

Liu B, Wang D, Yu G, Meng X (2013) Removal of F− from aqueous solution using Zr(IV) impregnated dithiocarbamate modified chitosan beads. Chem Eng J 228:224–231

https://doi.org/10.1016/j.cej.2013.04.099

Liu J, Yue X, Lu X, Guo Y (2018) Uptake fluoride from water by starch stabilized layered double hydroxides. Water 10:745

https://doi.org/10.3390/w10060745

Liu M, Zang Z, Zhang S, Ouyang G, Han R (2021) Enhanced fluoride adsorption from aqueous solution by zirconium (IV)-impregnated magnetic chitosan graphene oxide. Int J Biol Macromol 182:1759‒1768

https://doi.org/10.1016/j.ijbiomac.2021.05.116

Ma J, Shen Y, Shen C, Wen Y, Liu W (2014) Al-doping chitosan–Fe(III) hydrogel for the removal of fluoride from aqueous solutions. Chem Eng J 248:98–106

https://doi.org/10.1016/j.cej.2014.02.098

Ma W, Lv T, Song X, Cheng Z, Duan S, Xin G, Liu F, Pan D (2014) Characteristics of selective fluoride adsorption by biocarbon-Mg/Al layered double hydroxides composites from protein solutions: Kinetics and equilibrium isotherms study. J Hazard Mater 268:166‒176

https://doi.org/10.1016/j.jhazmat.2014.01.013

Mahmoud ME, Abdelfattah AM, Tharwat RM, Nabil GM (2020) dsorption of negatively charged food tartrazine and sunset yellow dyes onto positively charged triethylenetetramine biochar: Optimization, kinetics and thermodynamic study. J Mol Liq 318:114297

https://doi.org/10.1016/j.molliq.2020.114297

Meng S, Yao Z, Liu J, Wang E, Li C, Jiang B, Xu Z (2022) Carbon dots capped cerium oxide nanoparticles for highly efficient removal and sensitive detection of fluoride. Journal of Hazardous Materials 435:128976

https://doi.org/10.1016/j.jhazmat.2022.128976

Mohamed R, El-Maghrabi HH, Riad M, Mikhail S (2017) Environmental friendly FeOOH adsorbent materials preparation, characterization and mathematical kinetics adsorption data. J Water Process Eng 16:212–222

https://doi.org/10.1016/j.jwpe.2017.01.005

Mohammadi M, Ameri-Shahrabi MJ, Sedighi M (2012) Comparative study of linearized and non-linearized modified Langmuir isotherm models on adsorption of asphaltene onto mineral surfaces. Surf Eng Appl Elect 48:234243

https://doi.org/10.3103/S1068375512030088

Mondal AK, Mandal B, Mandal LN (1993) Boron Adsorption characteristics of some acidic alluvial soils in relation to soil properties. Commun Soil Sci Plan 21:2553–2567

10.1080/00103629309368977

Nagaraj A, Sadasivuni KK, Rajan M (2017) Investigation of lanthanum impregnated cellulose, derived from biomass, as an adsorbent for the removal of fluoride from drinking water. Carbohyd Polym 176:402‒410

https://doi.org/10.1016/j.carbpol.2017.08.089

Pandi P, Viswanathan N (2014) Synthesis of alginate bioencapsulated nano-hydroxyapatite composite for selective fluoride sorption. Carbohyd Polym 112:662‒667

https://doi.org/10.1016/j.carbpol.2014.06.029

Sadhu M, Bhattacharya P, Vithanage M, Sudhakar PP (2022) Adsorptive removal of fluoride using biochar – A potential application in drinking water treatment. Sep Purif Technol 278:119106

https://doi.org/10.1016/j.seppur.2021.119106

Sahoo SK, Hota G (2018) Surface functionalization of GO with MgO/MgFe2O4 binary oxides: A novel magnetic nanoadsorbent for removal of fluoride ions. J Environ Chem Eng 6:2918–2931

https://doi.org/10.1016/j.jece.2018.04.054

Sivasankara V, Murugesh S, Rajkumar S, Darchen A (2013) Cerium dispersed in carbon (CeDC) and its adsorption behavior: A first example of tailored adsorbent for fluoride removal from drinking water. Chem Eng J 214:45‒54

https://doi.org/10.1016/j.cej.2012.10.023

Sujana MG, Pradhan HK, Anand S (2009) Studies on sorption of some geomaterials for fluoride removal from aqueous solutions. J Hazard Mater 161:120‒125

https://doi.org/10.1016/j.jhazmat.2008.03.062

Teng SX, Wang SG, Gong WX, Liu XW, Gao BY (2009) Removal of fluoride by hydrous manganese oxide-coated alumina: Performance and mechanism. J Hazard Mater 168:1004‒1011

https://doi.org/10.1016/j.jhazmat.2009.02.133

Vences-AlvarezaLitz E, Velazquez-Jimenez H, Chazaro-Ruiz LF, Diaz-Flores PE, Rangel-Mendez JR (2015) Fluoride removal in water by a hybrid adsorbent lanthanum–carbon. J Colloid Interf Sci 455:194‒202

https://doi.org/10.1016/j.jcis.2015.05.048

Wang M, Yu X, Yang C, Yang X, Lin M, Guan L, Ge M (2017) Removal of fluoride from aqueous solution by Mg-Al-Zr triple-metal composite. Chem Eng J 322:246–253

https://doi.org/10.1016/j.cej.2017.03.155

Wu T, Mao L, Wang H (2015) Adsorption of fluoride on Mg/Fe layered double hydroxides material prepared via hydrothermal process. RSC Adv 5:23246‒23254

https://doi.org/10.1039/C4RA16839A

Xie D, Gu Y, Wang W, Wang Y, Qin W, Wang G, Zhang H, Zhang Y (2019) Enhanced fluoride removal by hierarchically porous carbon foam monolith with high loading of UiO-66. J Colloid Interf Sci 542:269‒280

https://doi.org/10.1016/j.jcis.2019.02.027

Yang W, Tian S, Tang Q, Chai L, Wang H (2017) Fungus hyphae-supported alumina: An efficient and reclaimable adsorbent for fluoride removal from water. J Colloid Interf Sci 496:496‒504

https://doi.org/10.1016/j.jcis.2017.02.015

Yu X, Tong S, Ge M, Zuo J (2013) Removal of fluoride from drinking water by cellulose@hydroxyapatite nanocomposites. Carbohyd Polym 92:269‒275

https://doi.org/10.1016/j.carbpol.2012.09.045

Yu Y, Yu L, Chen JP (2015) Adsorption of fluoride by Fe–Mg–La triple-metal composite: Adsorbent preparation, illustration of performance and study of mechanisms. Chem Eng J 262:839–846

https://doi.org/10.1016/j.cej.2014.09.006

Zhang J, Chen N, Tang Z, Yu Y, Hu Q, Feng C (2015) A study of the mechanism of fluoride adsorption from aqueous solutions onto Fe-impregnated chitosan. Phys Chem Phys 17:12041‒12050

https://doi.org/10.1039/C5CP00817D

Zhang K, Wu S, He J, Chen L, Cai X, Chen K, Li Y, Sun B, Lin D, Liu G, Kong L, Liu J (2016) Development of a nanosphere adsorbent for the removal of fluoride from water. J Colloid Interf Sci 475:17‒25

https://doi.org/10.1016/j.jcis.2016.04.037

Zhang YZ, Jia Y (2016) Fluoride adsorption onto amorphous aluminum hydroxide: Roles of the surface acetate anions. J Colloid Interf Sci 483:295‒306

https://doi.org/10.1016/j.jcis.2016.08.054

Zhang YX, Ji Y (2016) Fluoride adsorption onto amorphous aluminum hydroxide: Roles of the surface acetate anions. J Colloid Interf Sci 483:295‒306

https://doi.org/10.1016/j.jcis.2016.08.054

Zhang C, Li Y, Wang TJ, Jiang Y, Fok J (2017) Synthesis and properties of a high-capacity iron oxide adsorbent for fluoride removal from drinking water. Appl Surf Science425:272‒281

https://doi.org/10.1016/j.apsusc.2017.06.159

Zhang T, Zhao B, Chen Q, Peng X, Yang D, Qiu F (2019) Layered double hydroxide functionalized biomass carbon fiber for highly efficient and recyclable fluoride adsorption. Appl Biol Chem 62:12

https://doi.org/10.1186/s13765-019-0410-z
